# Correlations of Electrophysiological Measurements with Identification Levels of Ancient Chinese Characters

**DOI:** 10.1371/journal.pone.0151133

**Published:** 2016-03-16

**Authors:** Zhengyang Qi, Xiaolong Wang, Shuang Hao, Chuanlin Zhu, Weiqi He, Wenbo Luo

**Affiliations:** 1 Research Center of Brain and Cognitive Neuroscience, Liaoning Normal University, Dalian, China; 2 Laboratory of Cognition and Mental Health, Chongqing University of Arts and Sciences, Chongqing, China; Southwest University, CHINA

## Abstract

Studies of event-related potential (ERP) in the human brain have shown that the N170 component can reliably distinguish among different object categories. However, it is unclear whether this is true for different identifiable levels within a single category. In the present study, we used ERP recording to examine the neural response to different identification levels and orientations (upright vs. inverted) of Chinese characters. The results showed that P1, N170, and P250 were modulated by different identification levels of Chinese characters. Moreover, time frequency analysis showed similar results, indicating that identification levels were associated with object recognition, particularly during processing of a single categorical stimulus.

## Introduction

We are exposed to words, just as we are exposed to faces, beginning in very early childhood. The effects of faces and words on our brain development and physiology are similar. Alphabet-based languages, such as English, do not include configural information, and we recognize the serial order of a restricted set of letters in different lengths[1]. In contrast, Chinese characters are similar to faces in what Chinese characters consist of radicals, similar to the eyes and nose of a face. Moreover, the meanings of Chinese characters (similar to that of different facial expressions) are understood through the long-term accumulation of experience. Therefore, Chinese characters share many properties with faces and are suitable for exploring visual word processing.

Currently, the study of words is based on prior studies of faces. Researchers have shown that some effects that were thought to be unique to faces can also be observed for words or even other objects. For example, the inversion effect, which was once considered to be evidence in support of face-specific processing[2–5], is also observed during recognition of words and other objects[6–8]. In particular, studies have shown behavioral indicators of the inversion effect, appearing as early as 130–200 ms on the surface of occipito-temporal cortex and peaking at the level of the N170 components in the event-related potential (ERP); when experts identify common objects[9], inverted faces and words elicited larger N170 amplitudes with longer latencies than objects. Moreover, functional magnetic resonance imaging (fMRI) has shown that the visual word form area (VWFA) in the left fusiform gyrus may overlap with the pre-existing fusiform face area (FFA)[10]. Accumulating evidence has shown that object processing, particularly face processing, tends to be performed as a type of expertise processing. Gauthier et al. tested bird and car experts with fMRI and found that the level of categorization and expertise, rather than superficial properties of objects, determine the specialization of the FFA[11]. In addition to common objects experts, Gauthier et al. created Greebles (a type of face-like artificial object) and trained participants to be experts at identifying a set of Greebles. The overall results showed that the processing methods for recognition of faces versus identification of Greebles were different among novices but similar among experts. In particular, a mechanism similar to the inversion effect was observed at the behavioral and ERP levels[12–14]. Several expertise markers in face processing have been identified, including holistic processing, and humans are all considered experts at facial recognition based on our extensive exposure to faces since birth.

In contrast, research results of Chinese characters are not in agreement and do not directly show expertise processing. Some researchers have reported a face-like N170 response elicited by Chinese characters, with an obvious right hemisphere advantage[7]. Thus, these results partly suggest that visual expertise processing occurs; if this is true, individuals who are more familiar with Chinese characters should exhibit larger N170 responses than those less familiar with Chinese characters. However, in several studies, the amplitude of N170 evoked by Chinese characters was significantly higher in 7-year-old participants than that in 11-year-old participants and adults[15]. Additionally, Fan et al. found that the N170 component is modulated by the observer’s symbol identification proficiency level, and N170 changes reflect competition between faces and identifiable characters[16]. Two papers also found different lateralization of Chinese characters. Wang et al. (2011) reported right hemisphere lateralization of Chinese characters, which was similar to faces and considered to be related to holistic processing, while Weng et al. (2011) reported that Chinese characters were left-lateralized, similar to English words[6]. Another separate study should that Chinese characters elicited a bilateral effect. Studies of emotional adjectives and nouns reported a left-lateralized hemisphere, consistent with the traditional understanding of language laterality. Brain lateralization involves large amounts of information, and studies have not been consistent in this regard. Thus, the mechanisms mediating visual word processing are not yet clear.

In the current study, we examined the correlation of electrophysiological measurements with the recognition of ancient Chinese characters. We selected Xiaozhuan font characters as experimental materials; these characters, which are generally unknown, thereby allowing participants to be trained to distinguish among the specific characters, are pictographs, which have been shown to be processed as characters[8]. In addition, we added orientation factor to investigate whether there was an inversion effect on Xiaozhuan font characters and to determine whether this effect different from the results of previous research with other modern Chinese characters.

## Methods

### Study design

In this study, we examined electrophysiological measurements during the recognition of ancient Chinese characters using Xiaozhuan font characters. We observed the processing of ancient Chinese characters using the ERP technique. This result uses the across-trial average of several electroencephalographic (EEG) traces. Importantly, in addition to the analysis of time- and phase-locked deflections (i.e., ERPs), we also measured transient modulations of the ongoing oscillatory EEG activity. These modulations may be reflected by an increase (event-related synchronization [ERS]) or decrease (ERS) in EEG power, not phase-locked to the stimulus onset and usually confined to a specific frequency range. Depending on their frequencies, ERS and ERD may represent neuronal mechanisms involved in cortical activation, inhibition, and binding[17]. Therefore, investigating these changes may reveal novel neural mechanisms that are involved in the ancient Chinese characters processing.

### Subjects

A total of 20 right-handed volunteers (9 males, 11 females; age = 21–26 years; average = 22.1 years) from Dalian, China, took part in this study. They reported themselves as being healthy, without any neurological and psychiatric disorders, and had normal or corrected-to-normal visual acuity. The experiment included training part and formal experiment, and participants must attend two parts at the same time, of course they got financial compensation for their participation. All subjects were provided informed written consent prior to the study. The study was approved by Liaoning Normal University Human Research Institutional Review Board in accordance with the Declaration of Helsinki (1991).

### Stimuli

We used 30 Xiaozhuan font characters in this study. In order to avoid the migration effect, these 30 Xiaozhuan font characters were completely distinct in terms of stroke type. Among 30 characters, 10 characters were adopted as distracters in the training part, and the remaining 20 characters were used in the formal experiment: 10 characters could be identified by participants after training, and 10 characters could not be identified by participants without training. Images of stimuli were white characters on a black background. The images were processed with MATLAB 2012a software to ensure that they measured 7.5 cm wide (equal to 3.77° when viewed 114 cm from the monitor) and 8.5 cm long. The orientation of the characters was upright or inverted.

### Procedure

#### Training

All participants recruited in this study had no experience in calligraphy. Therefore, we trained them to identify 10 Xiaozhuan font characters. In order to balance the physical stimuli, 10 participants studied 10 Xiaozhuan font characters, while the other 10 participants studied another 10 Xiaozhuan font characters. Participants were trained three times altogether. They practiced writing every character and were trained to understand the meaning of each character. We then used three methods for testing the success of the training sessions after the participants reported having learned their 10 characters: 1) a recall task, which required participants to write the Song font characters corresponding to the Xiaozhuan font characters accurately and fluently; 2) an opposite recall task, in which participants were required to write the Xiaozhuan font characters according to the Song font characters correctly and quickly; and 3) a recognition task, in which participants judged whether they had learned the presented Xiaozhuan font character (10 characters were trained, another 10 characters were distractors), presented in the upright or inverted form for 2 s. The accuracy in this task was above 95%. Secondly, if participants completed the three tasks, they performed all tasks again after 30 min to ensure that they had fully learned the 10 characters. Participants performed the three tasks a third time before the formal experiment. We excluded one person who was not up to the standard.

#### Formal experiment

**Fig 1:**
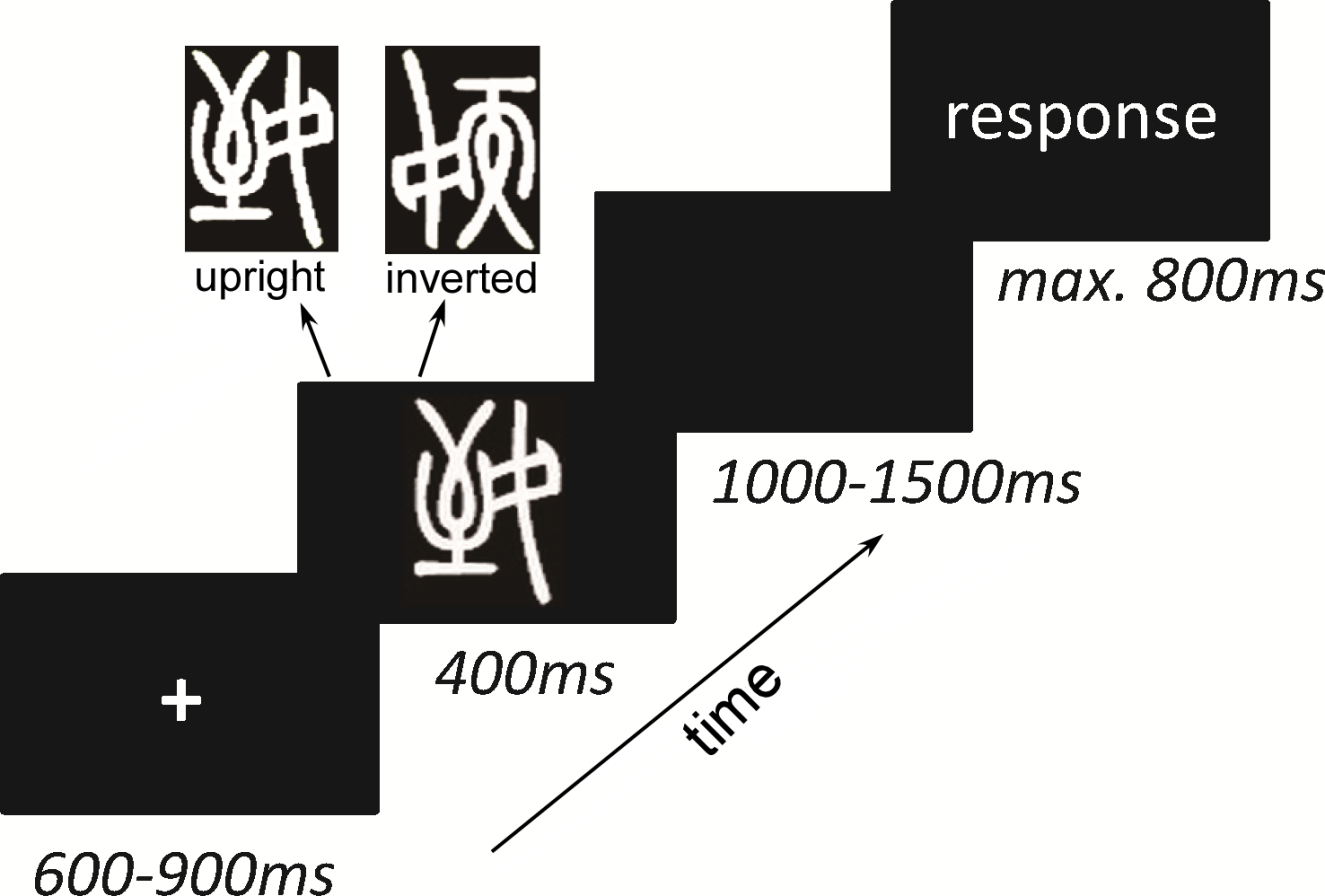
Schematic representation of experimental procedure. The Xiaozhuan-font characters, either learned or not, were presented in an upright or inverted form. Participants were required to judge whether they had learned the presented character or not by pressing “1” (learned) or “2” (not learned).

Participants were comfortably seated in a sound-isolated, dim room 114 cm away from a monitor (according to the center of the screen). The overall experimental procedure included four blocks, and each block had 80 trials. The trial was composed of a fixation, during which a blank screen was presented for 600–900 ms, followed by a Xiaozhuan font character for 400 ms in an upright or inverted form and then a blank screen for 1000–1500 ms to avoid the expecting effect. Finally, the response page was presented for 800 ms (Fig 1). Participants were asked to distinguish whether they had learned the presented character or not by pressing the keys when the response page appeared. Participants pressed “1” for characters they had learned and “2” for characters they had not learned.

### Behavioral data analysis

A two-way repeated-measures analysis of variance (ANOVA) was performed on behavioral data (mean reaction time and accuracy) to test the effects of ‘Identification’ (Identifiable vs. Unidentifiable) and ‘Orientation’ (Upright vs. Inverted).

### EEG recording

EEG data were recorded by a 64-channel system composing of tin electrodes mounted in an elastic cap (Brain Product, Germany) according to the international 10–20 System, with Cz reference. Horizontal electrooculography (EOG) was measured by an electrode placed on the outer canthi of right eye, and vertical EOG was measured by an electrode fixed on the infra-orbital ridge of left eye. Impedance of all electrodes was kept below 5kΩ. EEG and EOG were continuously recorded with a sampling rate of 500 Hz, filtered via a 0.01–100 Hz bandpass.

### EEG data analysis

#### Analysis in the time domain

EEG data were analyzed using EEGLAB [18]. Continuous EEG data were filtered with a 0.1–30 Hz bandpass, re-referenced to a common average, and segmented into epochs of 1200 ms, including a 200 ms pre-stimulus baseline. After baseline correction, trials contaminated by gross artifacts were rejected when the amplitude of any electrode exceeded ±100 μV. Remaining EOG artifacts were corrected using a validated method base on independent component analysis (ICA) [19]. In all datasets, the independent components (ICs) related to eye movements had a large EOG channel contribution and a frontal scalp distribution. Trials, in which the subjects responded correctly, were kept for the following analysis.

**Fig 2:**
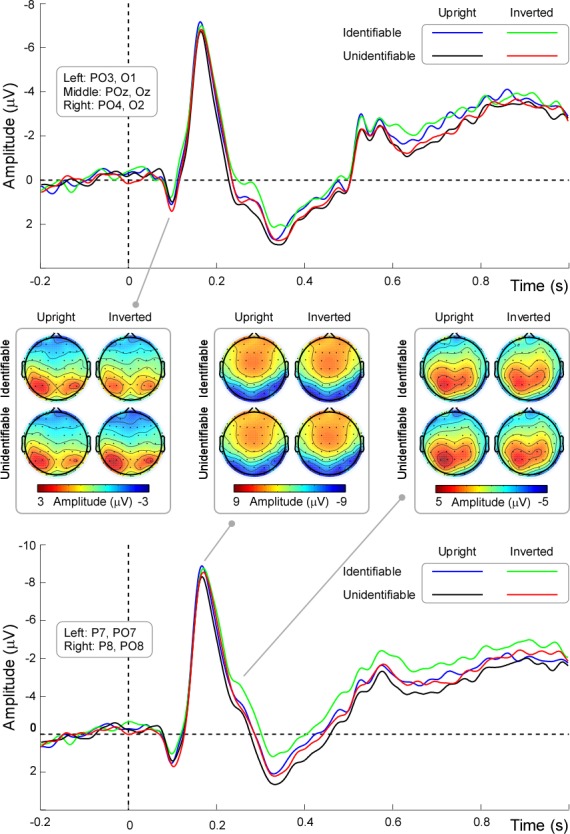
Group-level average ERP waveforms in different experimental conditions. ERP waveforms and scalp topographies of P1, N170, and P250 components are shown for each experimental condition. ERP waveforms are displayed in blue, green, black, and red for the experimental conditions using Identifiable-Upright, Identifiable-Inverted, Unidentifiable-Upright, and Unidentifiable-Inverted characters respectively. Top panel: Average ERP waveforms recorded at electrodes PO3, POz, PO4, O1, Oz and O2. Middle panel: Scalp topographies of P1, N170, and P250 components for all experimental conditions. Bottom panel: Average ERP waveforms recorded at electrodes P7, P8, PO7 and PO8. X axis, time (s); Y axis, amplitude (μV).

Based on the topographical distribution of grand-averaged ERPs (Fig 2) and previous studies [7, 20], a set of electrodes were chosen to measured P1, N170, and P250 components. P1 amplitude (86–112 ms) was measured from PO3, POz, PO4, O1, Oz, and O2 electrodes; N170 amplitude (150–180 ms) was measured from P7, P8, PO7, and PO8 electrodes; P250 amplitude (220-260ms) was analyzed at P7, P8, PO7, and PO8 electrodes. For each component, a three-way repeated-measures ANOVA was performed with the following variables as within-subject factors: ‘Hemisphere’ (three levels for P1: Left vs. Medial vs. Right; two levels for N170 and P250: Left vs. Right), ‘Identification’ (two levels: Identifiable vs. Unidentifiable), and ‘Orientation’ (two levels: Upright vs. Inverted). *p* value was corrected using the Greenhous-Geisser method.

#### Analysis in the time-frequency domain

The time-frequency distribution (TFD) of each single EEG trial was obtained using the windowed Fourier transform (WFT), in which a fixed Hanning window with a duration of 200 ms was used. For each single trial, the WFT translated EEG responses into a complex time-frequency spectral estimate with the explored frequencies ranging from 1 to 30 Hz in steps of 1 Hz, and the explored latencies ranging from -200 to 1000 ms in steps of 2 ms. For each estimated frequency, results were displayed as an event-related increase or decrease of oscillation amplitude relative to a pre-stimulus reference interval (-150 to -50 ms before the stimulus onset) using the unbiased subtraction approach [21].

**Fig 3:**
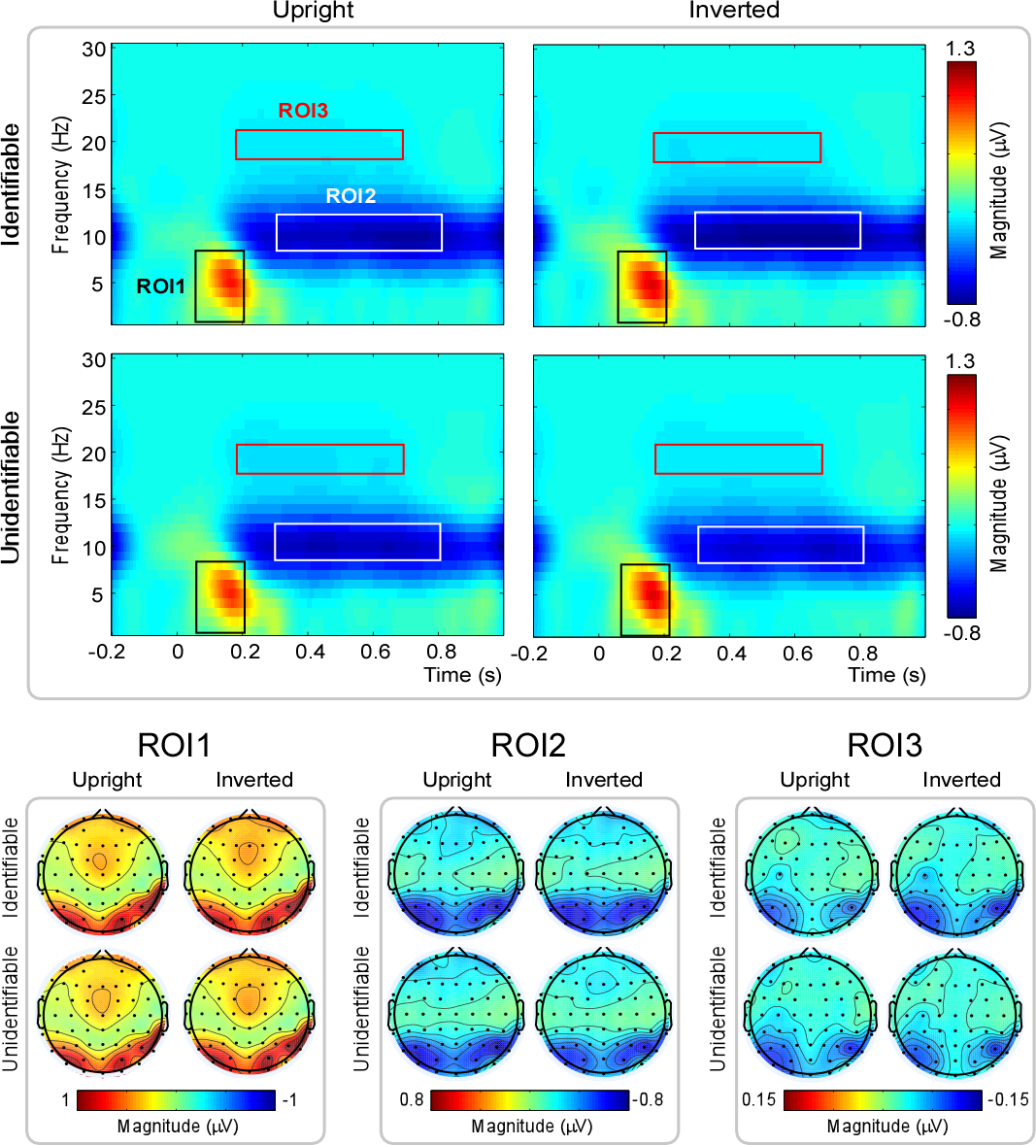
Grand-average time-frequency representations and scalp topographies in different experimental conditions. Top panel: Grand-average time-frequency representations are shown for each experimental condition. Three time-frequency region-of-interests (ROIs) were defined as follows: ROI1, delta-theta band oscillations [50–200 ms, 1–8 Hz, (P7 + PO3 + PO7 + P8 + PO4 + PO8)/6]; ROI2, alpha-band oscillations [300–800 ms, 8–13 Hz, (P1 + P3 + P2 + P4)/4]; ROI3, beta-band oscillations [200–700 ms, 17–21 Hz, (P3 + P5 + PO3 + P4 + P6 + PO4)/6]. Bottom panel: Scalp topographies of time-frequency oscillations in three ROIs for each experimental condition.

Across-trial averaging of time-frequency representations produced four average TFDs (one for each experimental condition: Identifiable-Upright, Identifiable-Inverted, Unidentifiable-Upright, Unidentifiable-Inverted) for each electrode and each subject. Three time-frequency region-of-interests (ROIs) were defined in the TFDs, where the oscillatory activities dominated. Also considering the topographical distribution of oscillatory activities (Fig 3), the time-frequency limits of each time-frequency ROI were as follows: ROI1, delta-theta band oscillation (1–8 Hz, 50–200 ms, measured from P7, P8, PO3, PO4, PO7, and PO8 electrodes), ROI2, alpha-band oscillation (8–13 Hz, 300–800 ms, measured from P1, P2, P3, and P4 electrodes), ROI3, beta-band oscillation (17–21 Hz, 200–700 ms, measured from P3, P4, P5, P6, PO3, and PO4 electrodes). For each ROI, oscillatory magnitude was obtained by calculating the mean magnitude within the pre-defined ROI [22].

For the oscillatory magnitude within each ROI, a three-way repeated measures ANOVA was performed with the following variables as within-subject factors: ‘Hemisphere’ (two levels: Left vs. Right), ‘Identification’ (two levels: Identifiable vs. Unidentifiable); and ‘Orientation’ (two levels: Upright vs. Inverted). *p* value was corrected using the Greenhous-Geisser method.

## Results

Means and standard deviation (mean ± SD) of behavioral data at different experimental conditions are reported in Table 1. Means and standard errors (mean ± SE) of electrophysiological data at different experimental conditions are reported in Table 2.

**Table 1:** Accuracy (mean ± SD) and reaction time (mean ± SD) at different experimental conditions.

	Accuracy (% correct)	Reaction time (ms)
**Identifiable -Upright**	98.80 ± .02	299 ± 46
**Identifiable -Inverted**	99.00 ± .02	297 ± 41
**Unidentifiable -Upright**	98.40 ± .02	297 ± 47
**Unidentifiable -Inverted**	98.3 ± .02	297 ± 48

**Table 2:** Amplitudes (mean ± SE) of electrophysiological parameters (P1, N170, and P250 components, as well as delta-theta band, alpha-band, and beta-band oscillations) at different experimental conditions.

	P1 (μV)			N170 (μV)		P250 (μV)		delta-theta (μV)		alpha-band (μV)		beta-band (μV)	
	Left	Middle	Right	Left	Right	Left	Right	Left	Right	Left	Right	Left	Right
**Identifiable-Upright**	1.17 ± .62	.74 ± .80	.28 ± .63	-7.73 ± .94	-8.63 ± 1.41	-1.27 ± .96	-3.16 ± 1.25	.75 ± .16	1.25 ± .34	-.47 ± .16	-.51 ± .19	-.08 ± .02	-.07 ± .02
**Identifiable-Inverted**	.77 ± .64	.48 ± .69	-.04 ± .61	-7.08 ± .99	-8.89 ± 1.47	-1.96 ± .88	-3.98 ± 1.24	.78 ± .15	1.25 ± .33	-.53 ± .17	-.56 ± .17	-.10 ± .02	-.09 ± .02
**Unidentifiable-Upright**	1.20 ± .61	.59 ± .71	.29 ± .62	-7.03 ± .88	-8.19 ± 1.45	-.23 ± .93	-3.14 ± 1.30	.76 ± .14	1.12 ± .29	-.41 ± .13	-.49 ± .15	-.09 ± .02	-.09 ± .02
**Unidentifiable-Inverted**	1.58 ± .61	1.00 ± .65	.55 ± .58	-7.07 ± .99	-8.57 ± 1.48	-1.08 ± .98	-3.30 ± 1.30	.76 ± .16	1.24 ± .35	-.38 ± .15	-.44 ± .15	-.08 ± .02	-.08 ± .02

### Behavioral results

For accuracy, the main effects of ‘Identification’ [*F* (1, 19) = 3.05, *p* = .10, $\eta_{p}^{2}$ = .14] and ‘Orientation’ [*F* (1, 19) = .04, *p* = .85, $\eta_{p}^{2}$ = .002], as well as their interaction [*F* (1, 19) = .61, *p* = .45, $\eta_{p}^{2}$ = .03] were not significant. As for mean reaction time, trails with incorrect responses were excluded from the RT analysis. The main effects of ‘Identification’ [*F* (1, 19) = .05, *p* = .83, $\eta_{p}^{2}$ = .002] and ‘Orientation’ [*F* (1, 19) = .36, *p* = .55, $\eta_{p}^{2}$ = .02], as well as their interaction [*F* (1, 19) = .28, *p* = .60, $\eta_{p}^{2}$ = .02] were also not significant.

### Electrophysiological results in the time domain

We only average the correct trials, and valid trials for each condition are as follows: 71 valid trials for Identifiable-Upright condition, 69 valid trials for Identifiable-Inverted condition, 69 valid trials for Unidentifiable-Upright condition, and 69 valid trials for Unidentifiable-Inverted condition.

#### P1 component

**Fig 4:**
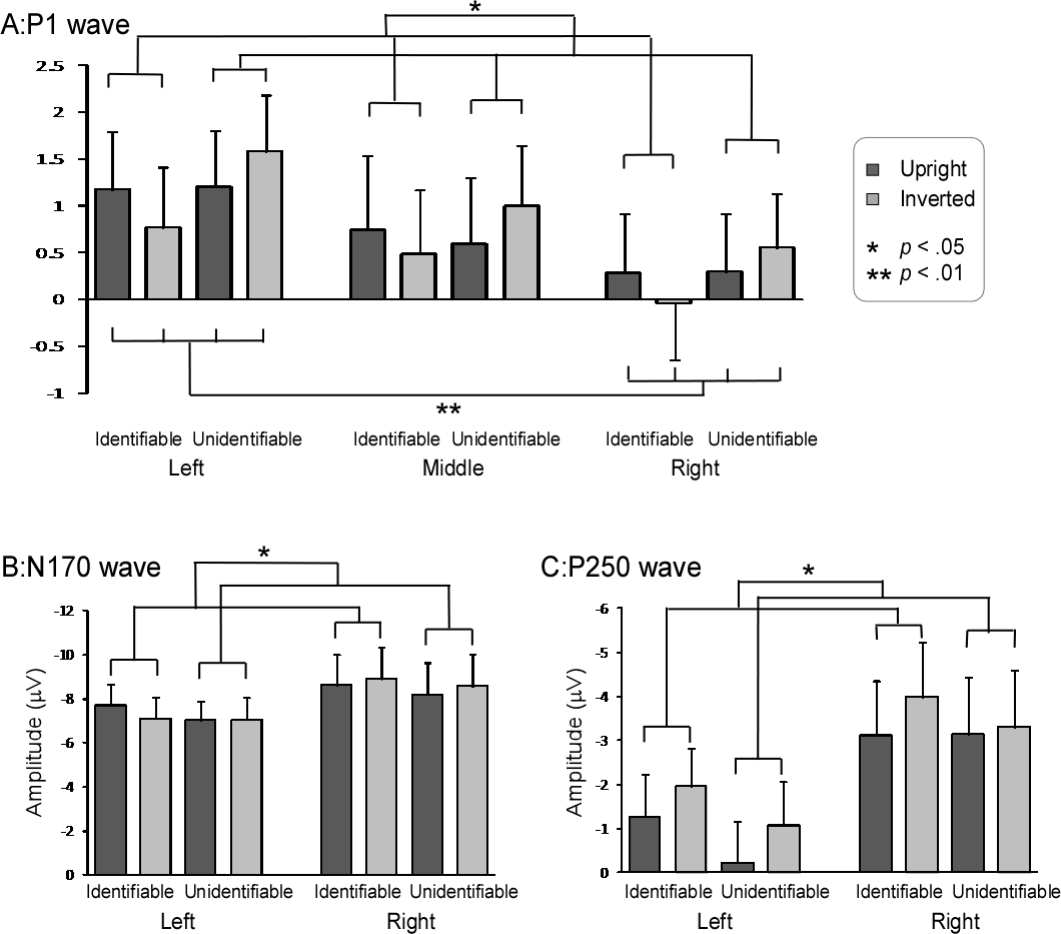
P1, N170 and P250 amplitudes in different experimental conditions. P1 amplitude was significantly larger in the left hemisphere than in the right hemisphere. While P1 amplitude was significantly smaller for Identifiable characters than Unidentifiable characters, both N170 and P250 amplitudes were significantly larger for Identifiable characters than Unidentifiable characters. Y axis, amplitude (μV, mean ± SE).

P1 amplitude showed significant main effects of ‘Hemisphere’ [*F* (2, 38) = 6.58, *p* = .007, $\eta_{p}^{2}$ = .26] and ‘Identification’ [*F* (1, 19) = 4.39, *p* = .05, $\eta_{p}^{2}$ = .19]. Post-hoc pairwise comparisons showed that P1 amplitude was significantly larger in the left hemisphere than in the right hemisphere (1.18 ± .60 μV vs. .27 ± .60 μV, *p* < .001) (Fig 4, panel A). In addition, Unidentifiable characters elicited significantly larger P1 amplitude than Identifiable characters (.87 ± .61 μV vs. .57 ± .64 μV, *p* = .05) (Fig 4, panel A).

There was a significant interaction between the effects ‘Identification’ and ‘Orientation’ [*F* (1, 19) = 8.15, *p* = .01, $\eta_{p}^{2}$ = .30]. Post-hoc simple effects analysis demonstrated that Unidentifiable-Inverted characters elicited significantly larger P1 amplitude than Unidentifiable-Upright characters (1.04 ± .59 μV vs. .69 ± .64 μV; *p* = .039). However, there was no significant difference between Identifiable-Upright characters and Identifiable-Inverted characters (.73 ± .67 μV vs. .40 ± .63 μV; *p* = .096).

#### N170 component

N170 amplitude showed a significant main effect of ‘Identification’ [*F* (1, 19) = 5.95, *p* = .025, $\eta_{p}^{2}$ = .24]. Post-hoc pairwise comparisons showed that Identifiable characters (-8.08 ± 1.13 μV) elicited significantly larger N170 amplitude than Unidentifiable characters (-7.72 ± 1.13 μV) (Fig 4, panel B).

In addition, there was a significant interaction between the effects ‘Hemisphere’ and ‘Orientation’ [*F* (1, 19) = 9.02, *p* = .007, $\eta_{p}^{2}$ = .32]. While there was no significant difference between N170 amplitudes elicited by Upright characters and Inverted characters in the left hemisphere (*p* = .11) or in the right hemisphere (*p* = .19).

#### P250 component

P250 amplitude showed significant main effects of ‘Identification’ [*F* (1, 19) = 16.70, *p* = .001, $\eta_{p}^{2}$ = .47] and ‘Orientation’ [*F* (1, 19) = 16.26, *p* = .001, $\eta_{p}^{2}$ = .46]. Post-hoc pairwise comparisons showed that Identifiable characters (-2.59 ± .94 μV) elicited significantly larger P250 amplitude than Unidentifiable characters (-1.94 ± .98 μV) (Fig 4, panel C). In addition, Inverted characters (-2.58 ± .97 μV) elicited significantly larger P250 amplitude than Upright characters (-1.95 ± .95 μV).

There was a significant interaction between the effects ‘Hemisphere’ and ‘Identification’ [*F* (1, 19) = 13.69, *p* = .002, $\eta_{p}^{2}$ = .42]. Post-hoc simple effects analysis showed that Identifiable characters elicited significantly larger P250 amplitude than Unidentifiable characters both in the left (-1.61 ± .91 μV vs. -.65 ± .94 μV, *p* < .001) and right (-3.57 ± 1.24 μV vs. -3.22 ± 1.30 μV, *p* = .032) hemispheres. Additionally, the interaction of ‘Hemisphere’, ‘Identification’, and ‘Orientation’ [*F* (1, 19) = 8.74, *p* = .008, $\eta_{p}^{2}$ = .32] was significant. We went further and found there was a significant interaction of ‘Identification’ and ‘Orientation’ in the right hemisphere [F (1, 19) = 11.98, *p* = .003, $\eta_{p}^{2}$ = .39] showed that the Identifiable-Inverted characters elicited significantly larger amplitude than Identifiable-Upright characters in the right hemisphere (-3.98 ± 1.24 μV vs. -3.16 ± 1.25 μV, *p* = .001), while there was no significant difference between amplitude elicited by Unidentifiable-Inverted characters and Unidentifiable-Upright characters in the right hemisphere (-3.30 ± 1.30 μV vs. -3.14 ± 1.30 μV, *p* = .41). And there was no significant interaction of ‘Identification’ and ‘Orientation’ in the left hemisphere [F (1, 19) = .40, *p* = .54, $\eta_{p}^{2}$ = .21].

### Electrophysiological results in the time-frequency domain

#### ROI1: delta-theta band oscillations

**Fig 5:**
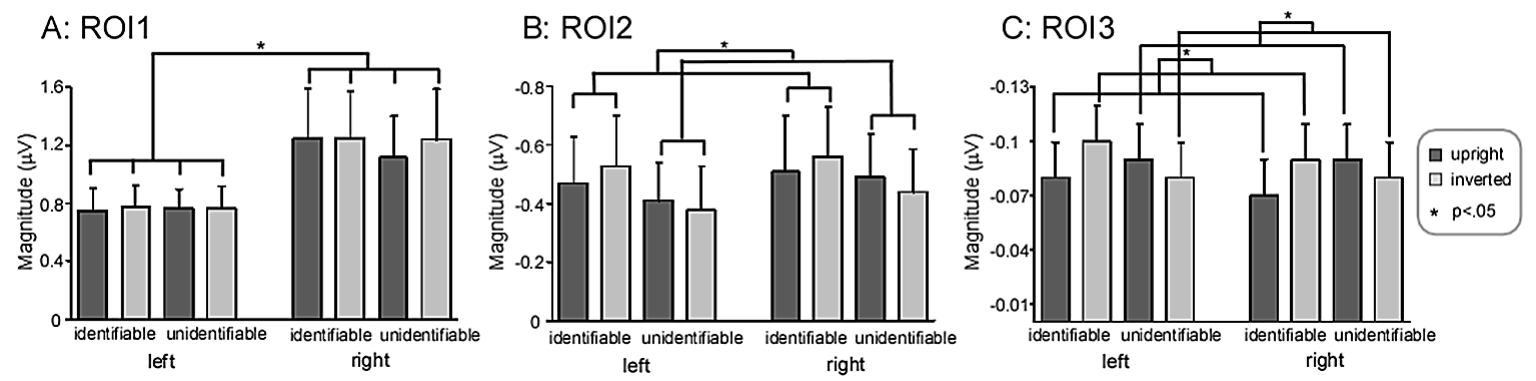
ROI magnitude in different experimental conditions. ROI1 magnitude was significantly larger in the right hemisphere than in the left hemisphere. ROI2 magnitude was significantly larger for Identifiable characters than Unidentifiable characters. ROI3 magnitude was significantly smaller for Identifiable-Upright characters than Identifiable-Inverted characters, and significantly larger for Unidentifiable-Upright characters than Unidentifiable-Inverted characters. Y axis, magnitude (μV, mean ± SE).

The magnitude of delta-theta band oscillations (ROI1) showed a significant main effect of ‘Hemisphere’ [*F* (1, 19) = 5.13, *p* = .035, $\eta_{p}^{2}$ = .21]. Post-hoc pairwise comparisons showed that ROI1 magnitude was significantly larger in the right hemisphere (1.13 ± .33 μV) than in the left hemisphere (.76 ± .15 μV) (Fig 5, panel A).

There was a significant interaction between the effects ‘Hemisphere’, ‘Identification’, and ‘Orientation’ [*F* (1, 19) = 4.63, *p* = .045, $\eta_{p}^{2}$ = .20]. While we went further and found that there was no significant interaction of ‘Identification’ and ‘Orientation’ in the left hemisphere [F (1, 19) = .12, *p* = .74, $\eta_{p}^{2}$ = .006] or right hemisphere [F (1, 19) = 1.24, *p* = .28, $\eta_{p}^{2}$ = .061].

#### ROI2: alpha-band oscillations

The magnitude of alpha-band oscillations (ROI2) showed a significant main effect of ‘Identification’ [*F* (1, 19) = 4.53, *p* = .046, $\eta_{p}^{2}$ = .19]. Post-hoc pairwise comparisons showed that Identifiable characters (-.52 ± .17 μV) evoked significantly lager magnitude than Unidentifiable characters (-.43 ± .14 μV) (Fig 5, panel B).

#### ROI3: beta-band oscillations

There was a significant interaction between the effects ‘Identification’ and ‘Orientation’ [*F* (1, 19) = 9.51, *p* = .006, $\eta_{p}^{2}$ = .33]. Post-hoc simple effects analysis showed that Identifiable-Inverted characters evoked significantly larger magnitude than Identifiable-Upright characters (-.10 ± .20 μV vs. -.08 ± .20 μV, *p* = .024), Unidentifiable-Upright characters evoked larger magnitude than Unidentifiable-Inverted characters (-.09 ± .20 μV vs. -.08 ± .20 μV, *p* = .047) (Fig 5, panel C).

## Discussion

In this study, using the study test pattern, we observed the neural mechanisms underlying different identification levels of ancient Chinese characters and their inversion effects. Unfortunately, we did not observe any significant differences in terms of accuracy or reaction time according to the level or identification and the orientation. This result may be explained by the simplicity of the task.

Our results can be summarized as follows. First, ERP components and alpha-band oscillations could be modulated by different identification levels of Chinese characters; unidentifiable characters elicited significantly larger P1 amplitude than identifiable characters, while identifiable characters elicited larger N170 and P250 amplitudes than unidentifiable characters. Additionally, identifiable characters evoked significantly larger magnitudes than unidentifiable characters on alpha-band oscillations. Additionally, the main effects on P250 amplitudes and the interaction appeared on P1 amplitudes, and differences in the orientations of beta-band oscillations reflected the inversion effect.

### The inversion effect of ancient Chinese characters

We observed that larger P250 amplitudes were elicited by inverted characters than upright characters, and unidentifiable inverted characters elicited larger P1 amplitudes than unidentifiable upright characters. Additionally, identifiable inverted characters elicited significantly larger magnitudes than identifiable upright characters in the beta-band ROI3. Although we did not observe an inversion effect on the N170 amplitudes, the inversion effect appeared on the P1 and P250 amplitudes and on beta-band oscillations, showing that the participants regarded the Xiaozhuan font characters as words rather than patterns and that the ancient Chinese characters had properties similar to those of modern Chinese characters.

The lack of the inversion effect may be explained by the influence of stimuli material itself. Although the configural information of Chinese characters is similar to that of faces, as previously described, Chinese characters are composed of radicals corresponding to the eyes and nose of a face; however, the relative positional information of radicals within Chinese characters is tighter than that within faces, and it is more difficult to process configural information for Chinese characters than for faces. Thus, the inversion effect of faces was more dramatic than that of Chinese characters. In particular, the stimuli used in the present study contained both visual and linguistic information. Xiaozhuan font characters are an ancient type of pictographic characters, and their structures are more complicated than modern Chinese characters; thus, these characters cannot be identified without proper training. Accordingly, the pre-existing differences between ancient characters and modern Chinese characters can be attributed to the unclear inversion effect of Xiaozhuan font characters.

Some researchers have examined the inversion effect of non-face stimuli, showing that the effects on N170 amplitude were left lateralized (for English words) or bilateral (for objects)[6]. However, Chinese characters and other logographic scripts[7, 23–25] do not elicit the typical left N170 lateralization observed for alphabetic scripts[6, 26]. For example, Wong et al. (2005) reported a left-lateralized response for Chinese characters and Chinese emotional nouns also showed a left-lateralized N170[27], while Wang et al. (2011) showed a right N170 amplitude lateralization associated with the inversion effect for compound Chinese characters. Similar to the experimental material used in our current study, pictographs have been shown to elicit bilateral N170 responses[8]. Although we used only Xiaozhuan font characters as stimuli material, the researchers in this previous report used three types of pictographic characters; however, we reached the same conclusion with Xiaozhuan font characters, showing bilateral N170 responses. Thus, the results of various studies on modern Chinese characters are inconsistent, and more studies are needed to elucidate these mechanisms. Our current results were consistent with the bilateral N170 effect of pictographs.

### ERP components and alpha-band oscillations are modulated by the identification level of Chinese characters

In the current study, we found that P1, N170, P250, and alpha-band oscillations induced by different identification levels of Chinese characters responded differently. The identifiable and unidentifiable characters in our study were designed to achieve expert- and novice-level object recognition[28, 29]. However, we cannot consider the participants to be experts in Xiaozhuan font characters because they can only recognize 10 characters. Thus, our result is similar to studies that found increased N170 amplitudes after object expertise training. In addition to object recognition, our results are also consistent with a previous study showing that N170 is sensitive to the character likeness of visual stimuli; faster, stronger N170 responses were observed for pictographs with a higher degree of character likeness. However, the participants in this previous study were unfamiliar with the pictographs, including the Xiaozhuan font characters and could not understand the meanings or sounds of the characters. Therefore, we do not know whether the participants actually understood the pictographs or whether they provided random guesses. In our study, we trained the participants three times, and the selected 30 Xiaozhuan font character were completely distinct in terms of stroke type; therefore, we ensured that the participants had a complete understanding of 10 of the characters but would not recognize the other 10 characters. Thus, our study strongly supported that identifiable Chinese characters can be modulated by the N170 component for visual information and the P250 component for visual semantic information.

Alpha-band oscillations are the dominant oscillations in the human brain. Recent evidence suggests that these oscillations have an inhibitory function. Modulation of the alpha-band magnitude reflects cortical arousal. An increased magnitude in the alpha frequency band indicates a lower state of arousal[30]. The general observation that an inward shift of attentional focus toward mental operations (as in mental rotation tasks, visual imagery, and other high-load working memory tasks) is typically accompanied by increases in posterior alpha power, suggesting that, in such cases, alpha might be working to inhibit sensory processing and decrease overall distractibility by sensory events[31]. Enhanced alpha power can reflect states of increased cognitive load. In our study, identifiable characters elicited more cognitive load than unidentifiable characters, which was similar to the results of ERP components.

Activity in the theta band may be related to a coordinated orienting response indicating alertness, arousal, and/or readiness to process information[32]. A previous study has indicated that event-related oscillations in the theta band may be correlated with selective attention[33]. Experimental data concerning event-related theta oscillations suggest that these oscillations have a basic role in cognitive processing[34, 35]. Event-related theta oscillations are observed after an inadequate stimulation, whereas event-related alpha oscillations are not existent if the stimulation is inadequate. Accordingly, the associative character for event-related theta oscillations is more pronounced than that for higher frequency event-related oscillations[35]. In our study, the delta-theta band oscillations corresponded to the combination of P1, N170, and P250. However, the ERP components had different phases, and the information of frequency of the delta-theta band was counteracted. We did not observe corresponding effects with P1, N170, or P250 components of Xiaozhuan font characters.

## Conclusion

In the current study, we used the ERP technique to investigate the correlations between electrophysiological measurements and different identification levels/orientations of ancient Chinese characters. Our findings were consistent with previous studies showing that the amplitude of the N170 component is modulated by the observer’s character identification level: identifiable Chinese characters elicited larger N170 amplitudes than unidentifiable characters. Furthermore, we also found that the N170, P1, and P250 components could be modulated by different identifiable Xiaozhuan font characters. The results of the time-frequency domain also supported our conclusion. Finally, we observed the effects of inversion on P250 and P1 components as well as beta-band oscillations. Overall, not only ERP components indicating time information are modulated by different identification levels, but frequency information also verifies this conclusion.
